# The complex relationship between body mass index and response to immune checkpoint inhibition in metastatic melanoma patients

**DOI:** 10.1186/s40425-019-0699-5

**Published:** 2019-08-19

**Authors:** Douglas Donnelly, Shirin Bajaj, Jaehong Yu, Miles Hsu, Arjun Balar, Anna Pavlick, Jeffrey Weber, Iman Osman, Judy Zhong

**Affiliations:** 1 The Ronald O. Perelman Department of Dermatology, New York University School of Medicine, New York, NY USA; 2 Laura and Isaac Perlmutter Cancer Center, NYU Langone Health, New York, NY USA; 3 Department of Population Health, NYU Langone Health, New York, NY USA; 4 Biostatistics, Epidemiology and Research Design Program (BERD), NYU-H+H Clinical and Translational Science Institute, 180 Madison Avenue, 4th Floor, Room 452, New York, NY 10016 USA

## Abstract

Despite major improvements in combatting metastatic melanoma since the advent of immunotherapy, the overall survival for patients with advanced disease remains low. Recently, there is a growing number of reports supporting an “obesity paradox,” in which patients who are overweight or mildly obese may exhibit a survival benefit in patients who received immune checkpoint inhibitors. We studied the relationship between body mass index and progression-free survival and overall survival in a cohort of 423 metastatic melanoma patients receiving immunotherapy, enrolled and prospectively followed up in the NYU Interdisciplinary Melanoma Cooperative Group database. We analyzed this association stratified by first vs. second or greater-line of treatment and treatment type adjusting for age, gender, stage, lactate dehydrogenase, Eastern Cooperative Oncology Group performance status, number of metastatic sites, and body mass index classification changes. In our cohort, the patients who were overweight or obese did not have different progression-free survival than patients with normal body mass index. Stratifying this cohort by first vs. non-first line immunotherapy revealed a moderate but insignificant association between being overweight or obese and better progression-free survival in patients who received first line. Conversely, an association with worse progression-free survival was observed in patients who received non-first line immune checkpoint inhibitors. Specifically, overweight and obese patients receiving combination immunotherapy had a statistically significant survival benefit, whereas patients receiving the other treatment types showed heterogeneous trends. We caution the scientific community to consider several important points prior to drawing conclusions that could potentially influence patient care, including preclinical data associating obesity with aggressive tumor biology, the lack of congruence amongst several investigations, and the limited reproduced comprehensiveness of these studies.

**Electronic supplementary material:**

The online version of this article (10.1186/s40425-019-0699-5) contains supplementary material, which is available to authorized users.

## Introduction

Despite major improvements in combatting metastatic melanoma (MM) since the advent of immunotherapy, the overall survival for patients with advanced disease remains low [1]. To optimize our therapeutic index, as treatment options continue to grow, it is imperative to identify clinical characteristics and/or biomarkers that are predictive of treatment response [2].

Obesity, defined as a body mass index (BMI) > 30 kg/m^2^, has conventionally been considered both a poor prognostic factor across most cancer types, and a preventable risk factor for many cancers. Specifically, multiple studies have linked obesity with increased likelihood of developing melanoma and with increased primary tumor thickness, a negative prognostic factor [3, 4]. More recently, there is a growing number of reports supporting an “obesity paradox,” in which patients who are overweight or mildly obese may exhibit a survival benefit, which is overcome at some undefined level of obesity [5–9].

McQuade et al. reported that in a cohort of MM patients, obese male patients treated with immune checkpoint inhibition (ICI) + dacarbazine or targeted therapy exhibited a survival benefit in multivariate analysis, compared to men with a normal BMI < 25 [5]. Most provocatively, the results demonstrated a linear relationship that did not reverse in patients with BMI ≥30 kg/m^2^. We believe that this study, and others published since then, have the potential to send a hastily premature message to patients and the oncologic research community of this rather complex relationship.

## Methods

We sought to study the relationship between BMI and progression-free survival (PFS) and overall survival (OS) in a cohort of 423 MM patients receiving ICI, enrolled and prospectively followed-up in the NYU Interdisciplinary Melanoma Cooperative Group database. Stage III and IV MM patients treated with ICI from 2003 to 2018 with known BMI at treatment initiation were classified as normal (< 25 kg/m^2^), overweight (25–29.9 kg/m^2^), obese (≥30 kg/m^2^). Patients’ best response was evaluated according to RECIST criteria, and data were recorded as complete response, partial response, stable disease, and progression of disease. Toxicity data was recorded using the Common Terminology Criteria for Adverse Events according to NIH/NCI guidelines.

### Statistical analysis

Baseline patient characteristics in each cohort were compared among the three BMI categories using the Chi square test (Table 1). Median and range of follow up time were calculated in the survivors. Kaplan–Meier curves were generated and compared by the log-rank test to estimate OS and PFS distribution for each BMI group. Using univariate and multivariable cox proportional hazard models, we analyzed the associations between BMI and PFS/OS, stratified by first vs. second or greater-line of ICI treatment. The multivariable analysis adjusted for age, gender, stage, lactate dehydrogenase (LDH), Eastern Cooperative Oncology Group performance status (ECOG PS), number of metastatic sites and BRAF mutation status (Tables 2 and 3). Separate models were performed for each treatment type (anti-CTLA-4, anti-PD-1, combination therapy).

**Table 1:** Baseline characteristics of Metastatic Melanoma Patients receiving Immune Checkpoint Inhibitors

		Normal Weight	Overweight	Obesity	Pvalue
N(%)	Total = 423	139 (33)	165 (39)	119 (28)	
Age (mean(SD))		62.1(16)	64.5(14)	66(13)	0.09
Gender	Male	75(54)	114(69)	78(66)	0.02
	Female	64(46)	51(31)	41(34)	
Stage	Stage III	11(8)	14(8)	15(13)	0.38
	Stage IV	128(92)	151(92)	104(87)	
ICI Treatment	CTLA-4	64(46)	75(45)	61(51)	0.65
	PD-1	49(35)	55(33)	41(34)	
	Combination	26(19)	35(21)	17(14)	
ECOG Performance Status	0	93(69)	120(75)	90(80)	0.18
	>=1	41(31)	40(25)	23(20)	
LDH	Normal	71(62)	97(69)	73(79)	0.02
	High	44(38)	44(31)	19(21)	
BRAF Mutation	V600	7(8)	9(8)	10(12)	0.06
	Other	0(0)	5(4)	0(0)	
	WT	84(92)	101(88)	74(88)	
Number of metastatic sites [mean (SD)]		2.6(1)	2.7(1)	2.3(1)	0.05
Line of TRT	First Line	87(63)	108(65)	77(65)	0.87
	Non-First Line	52(37)	57(35)	42(35)	
Alive Status	Alive	71(51)	77(47)	59(50)	0.74
	Dead	68(49)	88(53)	60(50)	
Follow up months [median (range)]		33.1(1.4-121.1)	38.6(2.7-172.2)	37.7(11.0-173.3)	0.52

**Table 2:** Univariate and Multivariable Cox Proportional Hazard Models of PFS vs BMI

		anti-CTLA-4		anti-PD-1		Combination	
		HR (95% CI)	*P* value	HR (95% CI)	*P* value	HR (95% CI)	*P* value
Univariate Model	Overweight (vs Normal BMI)	1.1(0.67,1.83)	0.7	1.58(0.78,3.19)	0.21	0.36(0.15,0.85)	0.02
	Obesity (vs Normal BMI)	0.98(0.59,1.63)	0.93	1.49(0.71,3.13)	0.3	0.17(0.04,0.65)	0.01
Multivariable Model	Overweight (vs Normal BMI)	0.97(0.56,1.69)	0.92	2.34(1.05,5.2)	0.04	0.5(0.15,1.71)	0.27
	Obesity (vs Normal BMI)	1.16(0.7,1.94)	0.57	2.46(1.03,5.89)	0.04	0.18(0.05,0.74)	0.02
	Female	0.94(0.59,1.51)	0.81	1.05(0.6,1.84)	0.85	1.8(0.72,4.49)	0.21
	Age at Treatment Initiation	1(0.98,1.01)	0.77	0.99(0.97,1.01)	0.28	0.99(0.95,1.03)	0.67
	Stage IV at Treatment Initiation(vs Stage III)	3(1.07,8.41)	0.04	0.88(0.3,2.64)	0.82	0.19(0.02,1.62)	0.13
	ECOG Status	1.78(1.22,2.6)	0.003	2.67(1.65,4.31)	0.003	3.02(1.44,6.32)	<.001
	LDH High	1.16(0.74,1.82)	0.52	2.25(1.08,4.7)	0.03	1.45(0.65,3.23)	0.36
	Number of Metastatic Sites	1.21(1.04,1.4)	0.02	1.11(0.91,1.36)	0.29	0.94(0.7,1.25)	0.68
	BRAF Mutated	0.86(0.37,2)	0.73	0.43(0.12,1.6)	0.21	1.83(0.61,5.54)	0.28

**Table 3:** Univariate and Multivariable Cox Proportional Hazard Models of OS vs BMI

		anti-CTLA-4		anti-PD-1		Combination	
		HR (95% CI)	*P* value	HR (95% CI)	*P* value	HR (95% CI)	*P* value
Univariate Model	Overweight (vs Normal BMI)	1.05(0.61,1.8)	0.86	1.29(0.7,2.36)	0.42	0.69(0.29,1.62)	0.39
	Obesity (vs Normal BMI)	1.07(0.63,1.8)	0.81	0.88(0.42,1.85)	0.74	0.57(0.19,1.73)	0.32
Multivariable Model	Overweight (vs Normal BMI)	0.83(0.47,1.47)	0.52	1.6(0.86,2.96)	0.14	0.7(0.2,2.47)	0.58
	Obesity (vs Normal BMI)	1.22(0.75,2)	0.42	1.04(0.48,2.24)	0.92	0.92(0.24,3.49)	0.9
	Female	0.73(0.45,1.18)	0.19	0.43(0.24,0.78)	0.01	1.18(0.48,2.92)	0.72
	Age at Treatment Initiation	1.01(0.99,1.02)	0.2	1.01(0.98,1.03)	0.61	1.01(0.98,1.04)	0.66
	Stage IV at Treatment Initiation(vs Stage III)	15.76(2.12,117.24)	0.01	1.07(0.38,2.98)	0.9	1.12(0.1,12.97)	0.93
	ECOG Status	2.23(1.62,3.07)	<.001	2.92(1.88,4.51)	<.001	2.45(1.47,4.1)	0.001
	LDH High	1.62(1.05,2.5)	0.03	2.35(1.27,4.35)	0.01	2.48(0.9,6.85)	0.08
	Number of Metastatic Sites	1.2(1,1.44)	0.06	1.16(0.97,1.38)	0.1	0.85(0.58,1.24)	0.4
	BRAF Mutated	0.99(0.53,1.85)	0.96	0.78(0.25,2.48)	0.68	0.78(0.19,3.16)	0.73

As an exploratory analysis, we further examined the association between the change in BMIs with the changes in patients’ responses and toxicity in the subset of MM patients who received multiple lines of ICI treatments. We first classified them as patients with constant, increased and decreased BMIs derived from the BMI classifications at the time of their later and earlier ICI treatment initiations. For patients with more than two lines of treatments, the last and first treatment lines were used. We then assessed their changes in ECOG PS, number of metastatic sites, best response and toxicity similarly between the lines of ICI treatments. We then used Fisher’s Exact Tests to assess the association between the change in BMIs with the changes in patients’ responses and toxicity.

## Results

As seen in Table 1, our cohort of 423 MM patients receiving ICI contained 139 (33%) patients with normal BMI; 165 (39%) patients with overweight BMI and 119 (28%) patients with obese BMI with a median follow up time of 36.3 months (1.4–173.3) since ICI treatment initiation. Three hundred forty-two (81%) patients in our cohort received treatment as part of standard of care, and the remaining patients were enrolled in a clinical trial. The baseline characteristics and follow up time of the MM cohort are distributed equally among patients in the three BMI classifications.

**Fig. 1:**
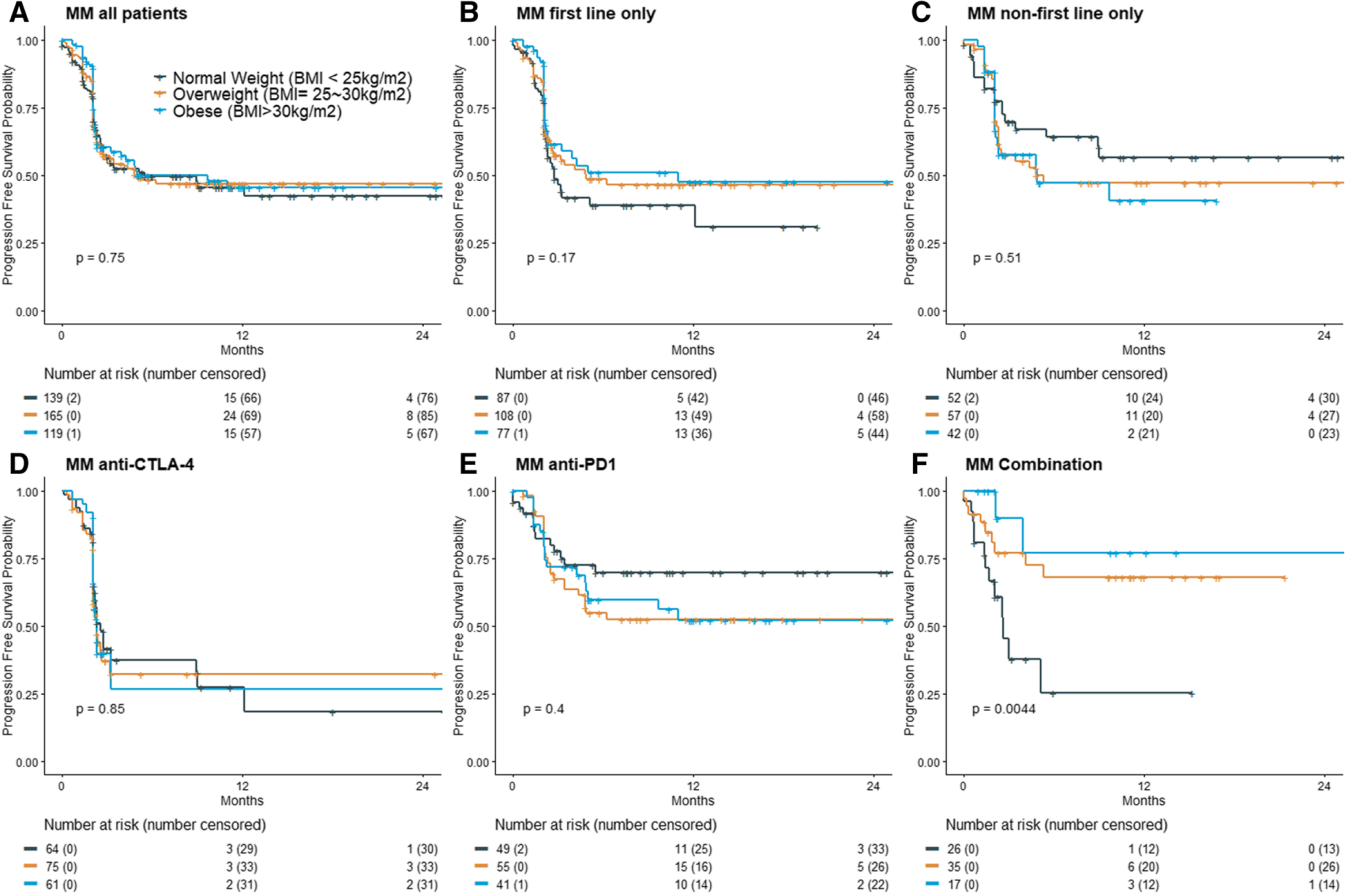
Progression-free survival by BMI shows heterogeneous trends when stratifying by clinical features. Progression-free survival in (**a**) the entire MM cohort, **b** the MM patients who received first-line ICI, **c** the MM patients who received non-first-line ICI, **d** the MM patients who received anti-CTLA4 treatment, **e** the MM who received anti-PD1 treatment, and (**f**) the MM patients who received combination treatment. All *p*-values are from the log-rank tests

**Fig. 2:**
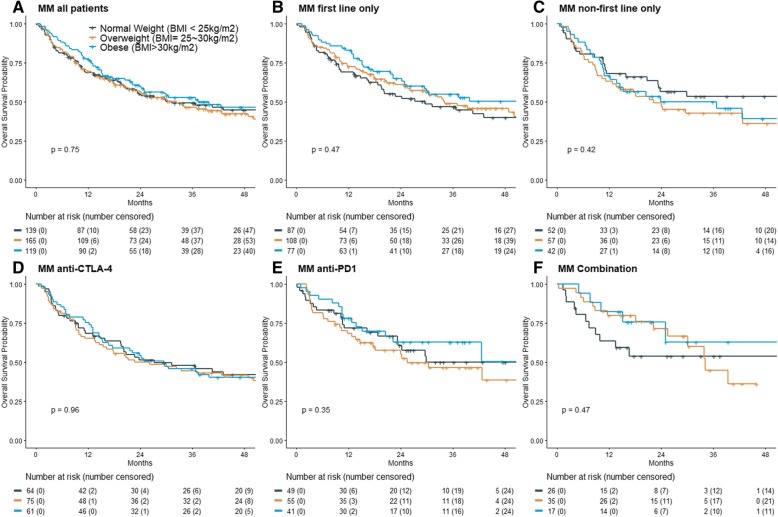
Overall survival by BMI shows no association between neither overweight nor obese classification. Overall survival in (**a**) the entire MM cohort, **b** the MM patients who received first-line ICI, **c** the MM patients who received non-first-line ICI, **d** the MM patients who received anti-CTLA4 treatment, **e** the MM who received anti-PD1 treatment, and **f** the MM patients who received combination treatment. All p-values are from the log-rank tests

Our MM patients treated with ICI who were overweight or obese did not have different PFS than patients with normal BMI, as seen in Fig. 1 (*P* = 0.75). Stratifying this cohort by first vs. non-first line ICI revealed a moderate but insignificant association between being overweight or obese and better PFS in patients who received first line ICI (*P* = 0.17). Conversely, an association with worse PFS was observed in patients who received non-first line ICI (*P* = 0.51). Figure 2 shows there was no OS benefit seen in patients that were overweight or obese (*P* = 0.75). Again, stratification by first vs. non-first line ICI showed a mild, yet insignificant association between overweight or obese BMI classifications and survival in first line ICI (*P* = 0.47), but it was reversed in the non-first line cohort (*P* = 0.42).

In a univariate analysis, overweight and obese patients receiving combination ICI had a statistically significant PFS benefit (HR = 0.36 [0·15–0.85]), *P* = 0.02 and HR = 0.17 [0.04–0.65, *P* = 0.01 for overweight and obesity groups respectively), whereas patients receiving the other treatment types showed heterogeneous trends (P interaction = .005). In the multivariable analysis, this significance held for obese patients (*P* = 0.02), but was lost in the overweight category (*P* = 0.27). In both univariate and multivariable analysis, no association was seen between increased BMI and OS in any ICI treatment (Tables 2 and 3). To examine robustness, we also performed univariate and multivariable models with all patients, stratified by treatment groups. There was no significant association between BMI and PFS or OS (Additional file 1: Table S1).

**Fig. 3:**
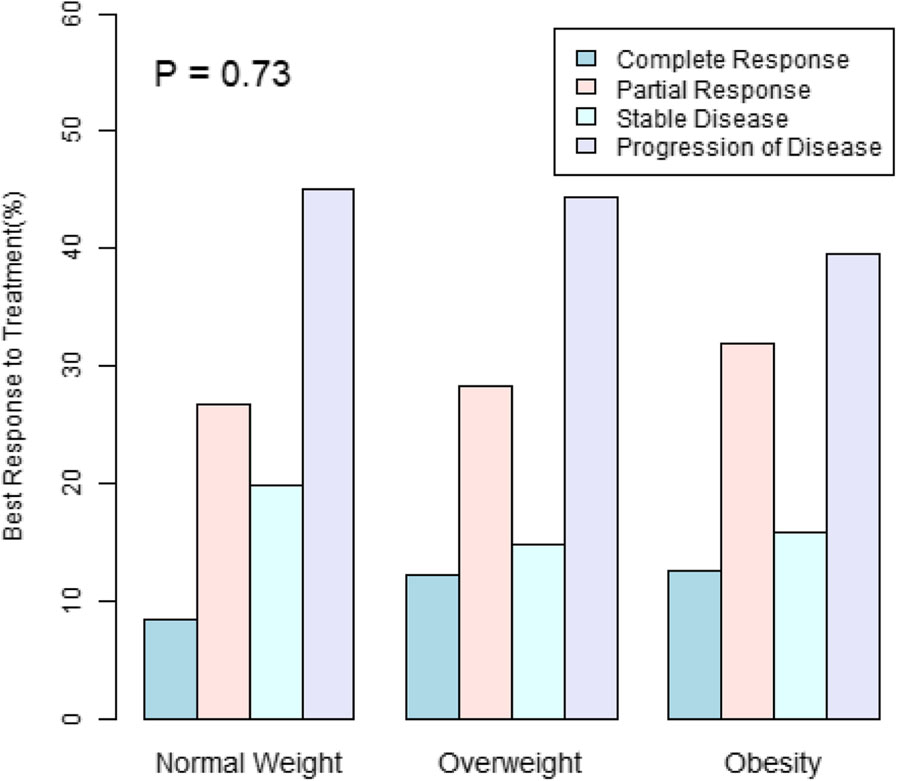
Best response is insignificantly associated with higher BMI. Best response percentages stratified by Normal Weight, Overweight, and Obesity

**Fig. 4:**
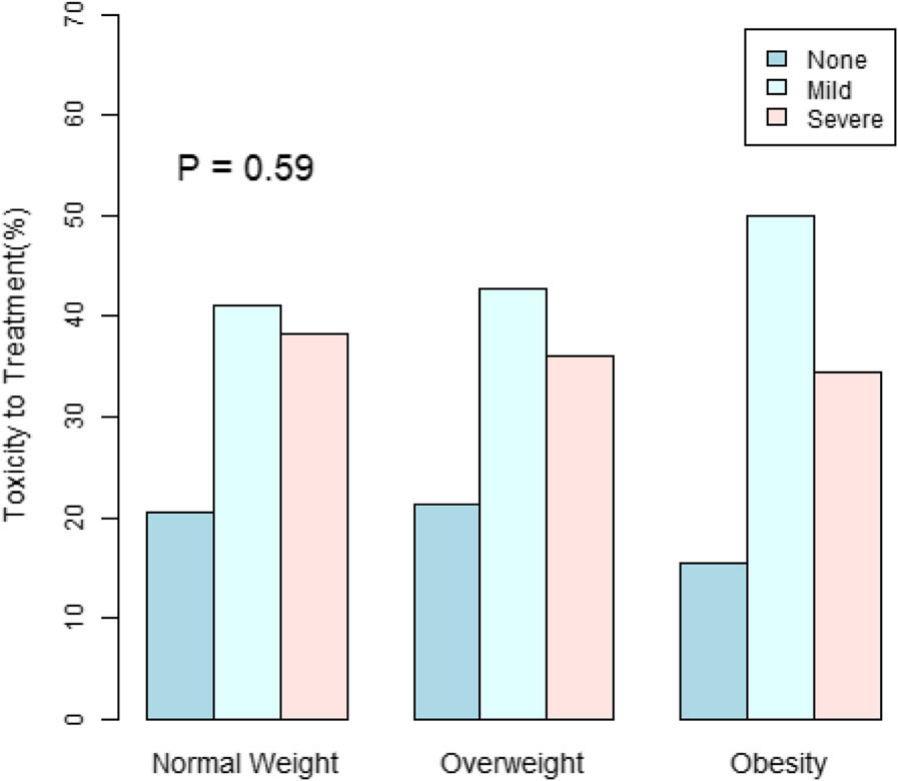
Toxicity is insignificantly associated with higher BMI. Immune-related adverse events stratified by Normal Weight, Overweight, and Obesity

Figures 3 and 4 reveal a positive association of BMI with best response and with higher rate of immune related adverse events. These associations, however, are not statistically significant.

Table 4 illustrates that 104 patients received multiple ICI treatment lines in our cohort. Of these patients, 86 (83%) patients remained constant in their BMI classifications, while 14 (13%) patients decreased from a higher to a lower BMI classification and 4 (4%) patients increased from a lower to higher BMI classification between multiple treatments. Decreases in BMI classification showed a trend of association with increases in ECOG PS and number of metastatic sites compared with patients with constant and increased BMIs. Interestingly, the four patients with increased BMIs during treatments showed higher percentages of deteriorating response categories (2/4) and decreased toxicities (3/4). Due to low sample numbers of patients who changed BMI categories during individual treatments, these results were insignificant and warrant further examination in larger cohorts.

**Table 4:** Association between BMI classification change and response/toxicity changes in patients receiving multiple

Change in BMI Classification		Higher to lower	Constant	Lower to Higher	*P* value
N(%)	Total = 104	14(13)	86(83)	4(4%)	
Change in ECOG Performance Status	Decrease PS	1(7)	3(3)	0(0)	0.41
	Constant PS	7(50)	61(71)	3(75)	
	Increase PS	6(43)	22(26)	1(25)	
Change in Number of metastatic sites	Decrease # of sites	1(7)	10(12)	2(50)	0.23
	Constant # of sites	4(29)	35(41)	1(25)	
	Increase # of sites	9(64)	41(48)	1(25)	
Change in Response	Improved OR	5(36)	30(35)	1(25)	0.08
	Constant OR	8(57)	24(28)	1(25)	
	Decrease OR	1(7)	32(37)	2(50)	
Change in Toxicity	Decrease Toxicity	4(29)	16(19)	3(75)	0.14
	Constant Toxicity	5(36)	37(43)	0(0)	
	Increase Toxicity	5(36)	33(38)	1(25)	

## Discussion

Our results, which showed heterogeneous trends when accounting for key clinical features, demonstrate the seemingly complex relationship between BMI and response to ICI. We caution the scientific community to consider several important points prior to drawing conclusions that could potentially influence patient guidance.

First, preclinical data strongly support the association between obesity and aggressive tumor biology across multiple species. Recent genetic and metabolic analyses of diet-induced obese mice bearing human B16 melanoma tumors demonstrated increased ulceration, tumor progression and invasion, and increased levels of PD-1 expression. In addition, analyses of publically available expression data cemented the association between obesity an immunosuppressed phenotype [6]. Patients with high BMIs are more likely to have chronic inflammation, which is associated with a decrease in M2 macrophages, CD8 T cells, and natural killer T cells [10]. Given these analyses, the mechanistic understanding to explain a possible survival benefit for obese patients receiving ICI is unclear. Future investigations should address baseline inflammation levels as well as sarcopenic vs. normal-weight obesity to better elucidate this mechanism.

Second, the pharmacokinetic characteristics of monoclonal antibody absorption, distribution, and clearance differ greatly from those of traditional small molecule drugs, as renal and biliary excretion is negligible [11]. Thus, mostly the liver must metabolize therapeutic antibodies prior to clearance. Obesity is associated with disturbances in metabolism via enhanced adipose secretion of free fatty acids and proinflammatory cytokines, which affect circulatory and hepatic functions [10, 11]. Further, studies have shown that body weight specifically influences the clearance and volume of distribution of therapeutic antibodies [11–13]. Given that dosing of ICI as weight-based vs. fixed has varied over time, across treatment types, and between institutions, the absence of pharmacokinetic control in analyzing the relationship between BMI and ICI represents another limitation in the generalizability of the results from this and prior studies. We hypothesize that there are underlying metabolic mechanisms that drive the observed positive association between BMI and response to ICI. To that end, metabolic profiles, created by combining host genomics, baseline inflammation and serum creatinine levels as indicators of adiposity, and tumor microenvironment features, may provide a more scientifically rational biomarker for response than BMI.

Finally, clinical data in support of a survival benefit are irreproducible amongst different investigations. Wang et al. and Naik et al. showed a significantly positive association between BMI and survival in melanoma patients treated with anti-PD1 ICI, McQuade et al. and Richtig et al. showed this positive association in patients treated with anti-CTLA4, and here we show the association in patients treated with combination anti-CTLA4 + anti-PD1 therapy [5, 6, 8, 9]. Notably, several investigations did not find the association in all treatment types analyzed in their studies. We acknowledge that the numerous positive reports suggest that BMI does influence response to ICI, but each of these studies utilized different covariates in their analyses as well as distinct statistical models to assess the associations. Furthermore, our cohort and other studied cohorts include patients treated as part of a clinical trial and as standard of care, which have different patient characteristics and outcomes due to inclusion criteria for clinical trials. These distinctions likely contribute additional complexity to the relationship. Moreover, an analysis of outcomes of 945 patients enrolled in phase III clinical trials demonstrated that the combination therapy had higher rates of PFS and OS than single-agent therapy in patients with BRAF mutations, stage M1c disease, and elevated LDH [14]. It is possible that our study is biased towards the null hypotheses due to lack of power or possible secular trends in melanoma diagnosis and ICI treatment effects in the prolonged study period [15]. This highlights the significance of systematically accounting for overall disease burden in the context of these and other variables while analyzing the association of BMI and response to ICI.

Several investigations have not reproducibly and comprehensively considered clinical confounders, such as genetic mutations, PD-L1 positivity, time-period of therapy and overall disease burden, which limits the ability to draw accurate conclusions about the relationship between BMI and response to ICI. When stratified by key clinical features including gender, treatment type, treatment line, tumor type, and BMI classification changes, our study and others have showed discordant results, and even reversal of trends. The varied designs and results amongst numerous studies highlight the need for a thorough investigation, on a significantly larger scale, to truly elucidate BMI’s impact on clinical outcomes in cancer. The scientific community must also pause to consider the known detrimental health effects of obesity, including hypertension, diabetes, and risk of cardiovascular disease when counseling patients that are treated with ICI [16, 17]. We believe it is of crucial importance to apply the same or higher level of scientific rigor to identifying an established negative health characteristic, such as high BMI, as a potential biomarker for a positive clinical outcome as the community applies to other biomarkers with limited preclinical data. Using previous publications to support original discoveries is central to the scientific method, but has the potential to over-emphasize perceived associations in cohort studies if numerous publications begin to cite inconclusive results.

## Additional file

Additional file 1: Univariate and multivariable analyses of all patients in study, not stratified by treatment type, demonstrate no significant association between BMI and PFS or OS. (PDF 9 kb)

## Data Availability

Some of the data generated or analyzed during this study are included in this published article. All additional data can be made available from the corresponding author on reasonable request.

## Abbreviations

BMI: Body mass index
ECOG PS: Eastern Cooperative Oncology Group performance status
ICI: Immune checkpoint inhibition
LDH: Lactate dehydrogenase
MM: Metastatic melanoma
OS: Overall survival
PFS: Progression-free survival
