# Supplementary material for: The complex relationship between body mass index and response to immune checkpoint inhibition in metastatic melanoma patients
Source: J Immunother Cancer. 2019 Aug 19;7:222. doi: 10.1186/s40425-019-0699-5 (PMC6700794; doi:10.1186/s40425-019-0699-5)
Supplement: Supplementary file 1 — Univariate and multivariable analyses of all patients in study, not stratified by treatment type, demonstrate no significant association between BMI and PFS or OS. (PDF 9 kb) [file 40425_2019_699_MOESM1_ESM.pdf]

		PFS		OS	
		HR (95% CI)	Pvalue	HR (95% CI)	Pvalue
Univariate Model	Overweight (vs Normal BMI)	1.02(0.68,1.52)	0.92	1.05(0.68,1.62)	0.82
	Obesity (vs Normal BMI)	0.9(0.6,1.35)	0.62	0.92(0.58,1.46)	0.73
Multivariable Model	Overweight (vs Normal BMI)	1.16(0.75,1.81)	0.5	1.05(0.67,1.66)	0.82
	Obesity (vs Normal BMI)	1.33(0.87,2.02)	0.19	1.2(0.73,1.99)	0.47
	Female	1.03(0.74,1.46)	0.85	0.64(0.41,1)	0.05
	Age at Treatment Initiation	1(0.99,1.01)	0.85	1(0.99,1.02)	0.54
	Stage IV at Treatment Initiation(vs Stage III)	1.63(0.73,3.65)	0.23	2.73(1.02,7.28)	0.05
	ECOG Status	1.94(1.44,2.61)	<0.001	2.28(1.76,2.96)	<0.001
	LDH High	0.75(0.51,1.1)	0.14	0.51(0.35,0.75)	<0.001
	Number of Metastatic Sites	1.58(1.03,2.42)	0.04	1.22(0.77,1.94)	0.4
	BRAF Mutated	1.13(1,1.28)	0.04	1.08(0.94,1.23)	0.29
